# Combined percutaneous auricular vagus nerve stimulation and ultrasound-guided phrenic nerve block for persistent hiccups: A case report

**DOI:** 10.1097/MD.0000000000049841

**Published:** 2026-07-17

**Authors:** Qinyue Luo, Guipeng Wu, Chuangang Shi, Zongfeng Guo, Jin Wang, Ran Wang, Xiaoqing Xu

**Affiliations:** a Department of Pain Medicine, Haian People’s Hospital of Nantong University, Haian, Jiangsu, China; b Department of Anesthesiology, Perioperative and Pain Medicine, Nanjing First Hospital, Nanjing Medical University, Nanjing, Jiangsu, China.

**Keywords:** auricular vagus nerve stimulation, case report, multimodal therapy, persistent hiccups, phrenic nerve block

## Abstract

**Rationale:**

Persistent hiccups lasting more than 48 hours are rare but can markedly impair quality of life. Conventional therapies are often limited by transient efficacy, side effects, invasiveness, highlighting the need for safer and more effective options.

**Patient concerns:**

A 62-year-old male with chronic hepatitis B, hypertension, type 2 diabetes, prior pulmonary tuberculosis, and gastrointestinal disorders presented with fever for 7 days and persistent hiccups for 4 days, occurring at a frequency of 40 to 50 times per minute. Symptoms significantly interfered with eating, sleeping, and daily activities.

**Diagnoses:**

After excluding central nervous system and metabolic causes, the patient was diagnosed with persistent hiccups associated with pulmonary infection and gastrointestinal dysfunction.

**Interventions:**

An initial ultrasound-guided phrenic nerve block provided transient relief for about 4 hours. Subsequently, combined therapy with daily phrenic nerve block and twice-daily percutaneous auricular vagus nerve stimulation (aVNS) was administered for 3 consecutive days.

**Outcomes:**

By day 7, the hiccups had resolved completely, with the hiccups assessment instrument score decreasing from 9 to 0 and the Pittsburgh sleep quality index score improving from 16 to 2. After initiation of aVNS, the patient was followed up for 1 month after discharge. No recurrence of persistent hiccups, delayed complications, or treatment-related adverse events were observed during follow-up.

**Lessons:**

This case suggests that ultrasound-guided phrenic nerve block combined with aVNS may offer a minimally invasive, safe, and effective treatment option for persistent hiccups unresponsive to conventional therapy. Larger studies are warranted to validate efficacy and long-term outcomes.

## 1. Introduction

Hiccups are involuntary, spasmodic contractions of the diaphragm and intercostal muscles, followed by sudden closure of the glottis, producing the characteristic sound.^[1]^ While most episodes are transient and self-limiting, persistent (lasting more than 48 hours) or intractable hiccups (lasting over 1 month) can significantly impair quality of life and often reflect underlying pathological processes, such as central nervous system lesions, gastrointestinal disorders, metabolic disturbances, or postoperative complications.^[2–4]^ The pathogenesis is thought to involve a “hiccup reflex arc” comprising vagal, phrenic, and sympathetic afferents T6–T12, central processing in the brainstem and cervical spinal cord, and phrenic efferent output to the diaphragm.^[5–8]^

Treatments for persistent or intractable hiccups can be broadly categorized into pharmacologic and non-pharmacologic approaches. Pharmacologic options (such as baclofen, gabapentin, chlorpromazine, and metoclopramide) often yield suboptimal efficacy with notable adverse effects, and high-quality evidence is lacking to identify a clearly superior agent.^[7,8]^ On the non-pharmacologic side, respiratory maneuvers (e.g., the Valsalva maneuver) and acupuncture typically provide only transient relief or no relief at all^[9–11]^; ultrasound-guided phrenic nerve block, C3–C5 nerve root block, or stellate ganglion block can promptly abort symptoms, but the benefits are usually short-lived and may require repetition^[12–14]^; implantable neuromodulation (phrenic or vagus nerve stimulation) may achieve more durable control but is invasive, poorly tolerated by some patients, and carries device-related risks such as lead migration.^[14,15]^ Here, we report the first case, to our knowledge, of combining percutaneous auricular vagus nerve stimulation (aVNS) with ultrasound-guided phrenic nerve block for persistent hiccups. This noninvasive multimodal approach provided complete and durable symptom relief and may represent a promising therapeutic option when conventional measures fail.

## 2. Clinical data

Written informed consent was obtained from the patient. The manuscript adheres to the Case Report guidelines, and all patient information has been anonymized.

### 2.1. Clinical background

A 62-year-old man was admitted with a 1-week history of fever and a 4-day history of persistent hiccups occurring at a frequency of 40 to 50 times per minute. The hiccups significantly interfered with eating, speaking, and sleep, and severely disrupted daily activities. His medical history was notable for chronic hepatitis B on long-term entecavir therapy, hypertension, type 2 diabetes mellitus, chronic atrophic gastritis, duodenal ulcer, prior pulmonary tuberculosis. He denied tobacco or alcohol use and had no prior episodes of persistent hiccups. The hiccups occurred in bursts, were more pronounced at night, and were occasionally accompanied by shortness of breath.

On admission, his temperature was 36.7°C, blood pressure 132/78 mm Hg, heart rate 78 bpm, respiratory rate 18 breaths/min, and oxygen saturation 98% on room air. He appeared visibly distressed, with reduced appetite, weight loss of approximately 2 kg over 1 week, and severe sleep fragmentation. His family also reported irritability and impaired social interaction. Gastrointestinal symptoms included abdominal distension, acid reflux, and belching. Physical examination revealed moist rales at the lung bases but no neurological deficits.

Laboratory findings showed leukocytosis (white blood cell 11.9 × 10^9^/L), hyperglycemia (17 mmol/L), and glycated hemoglobin 7.3%. Liver and renal function tests, as well as electrolytes, were within normal ranges. Magnetic resonance imaging of the brain and cervical spine excluded acute ischemia, tumors, or demyelinating disease. Chest-abdomen computed tomography demonstrated pulmonary consolidation, fibrosis, mild bronchiectasis, gastric wall thickening, and duodenal bulb lesions. Endoscopy confirmed chronic atrophic gastritis, esophagitis, and duodenitis.

Targeted therapy for pulmonary infection and gastrointestinal disorders led to clinical improvement, but hiccups persisted. Pharmacological treatments (including baclofen [10 mg 3 times daily for 3 day], gabapentin [300 mg twice daily], metoclopramide [10 mg 3 times daily], and promethazine [25 mg at night]) were ineffective. Traditional Chinese medicine (acupuncture and cupping) also failed to control symptoms. The patient was therefore referred to the pain management clinic for further intervention.

### 2.2. Intervention

#### 2.2.1. First intervention

**Figure 1.**
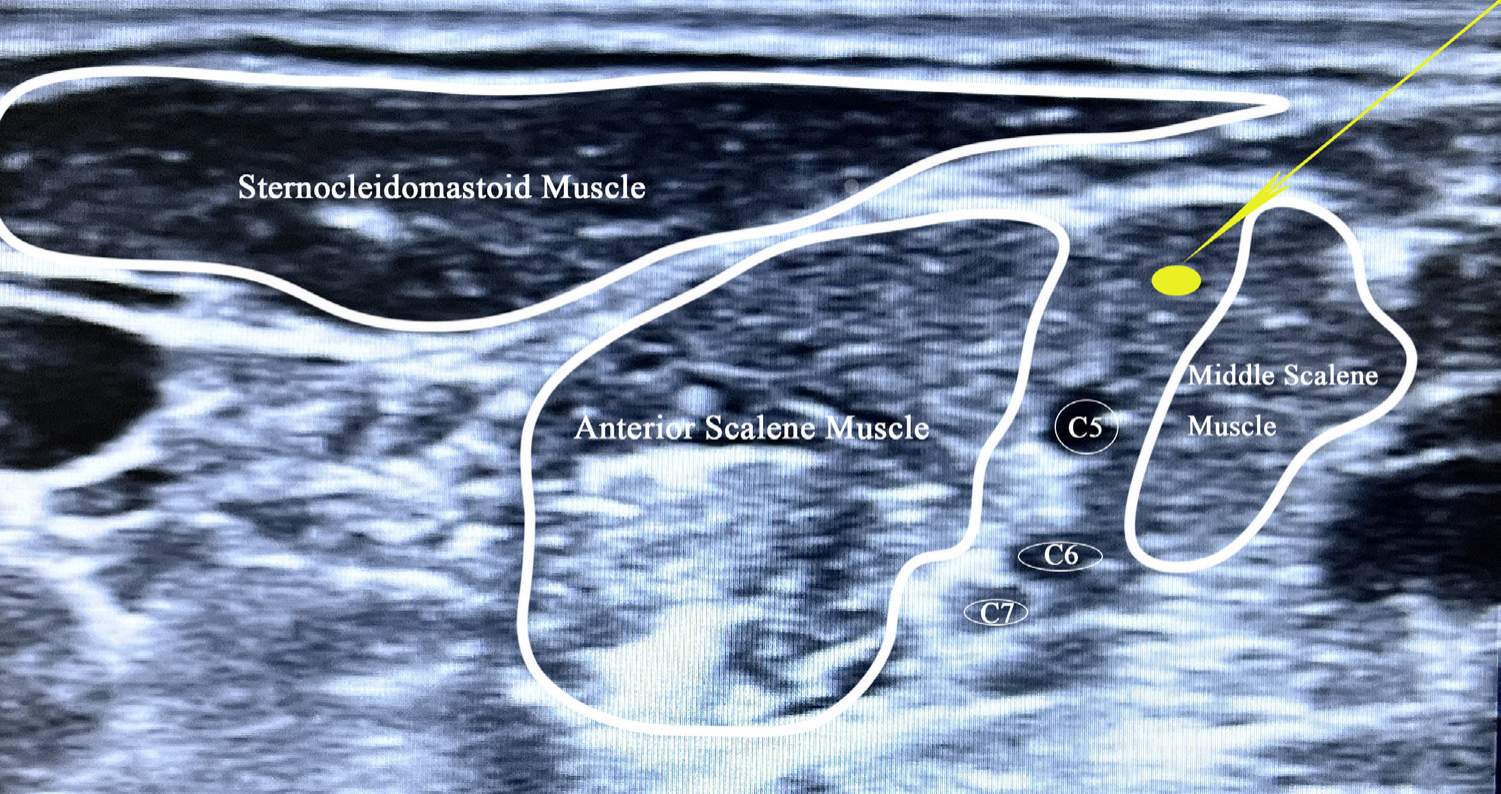
Ultrasound-guided phrenic nerve block puncture. The puncture needle (yellow arrow) is advanced toward the phrenic nerve (yellow dot) under ultrasound guidance. Surrounding anatomical landmarks, including the sternocleidomastoid, anterior scalene, and middle scalene muscles, are shown for reference. The use of this image has been approved by the patient with written informed consent.

The patient was placed supine without a pillow, head turned to the left. Vital signs were continuously monitored. A high-frequency linear ultrasound probe was positioned transversely at the junction of the middle and lower thirds of the posterior border of the sternocleidomastoid muscle. The phrenic nerve was identified as a hypoechoic oval fascicle encased in a hyperechoic sheath, located superior to the brachial plexus. After aseptic preparation, the needle was advanced under ultrasound guidance, avoiding vascular structures (Fig. 1) Negative aspiration confirmed no blood or cerebrospinal fluid. A total of 3 mL injectate (2% lidocaine 1.5 mL + saline 1.5 mL) was slowly administered. The needle was withdrawn, and local compression was applied. No dizziness or limb weakness occurred. Hiccups ceased immediately but recurred approximately 4 hours later as the local anesthetic effect subsided.

#### 2.2.2. Second intervention

**Figure 2.**
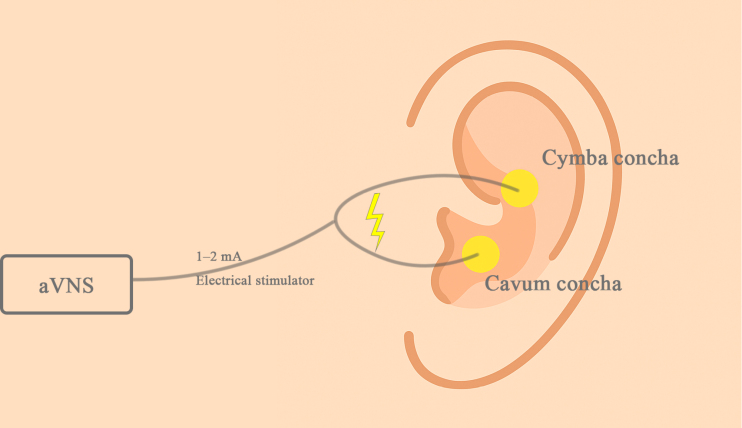
aVNS. The yellow dots represent the stimulation points. aVNS = auricular vagus nerve stimulation.

Given the short duration of benefit, combined therapy with ultrasound-guided phrenic nerve block and percutaneous aVNS was initiated (Fig. 2) Phrenic nerve blocks were performed once daily on alternate sides following the same technique as above. aVNS was applied by placing the electrode at the cymba concha and cavum concha. Stimulation was delivered at 20 Hz, 200 μs pulse width, 1 to 2 mA (maximum tolerated intensity), for 30 minutes per session, twice daily. Combined therapy was continued for 3 consecutive days.

Throughout all interventions, continuous electrocardiography, blood pressure, and pulse oximetry monitoring were performed. No bradyarrhythmias, hypotension, or respiratory compromise were observed. The patient tolerated stimulation without pain or skin irritation. His subjective feedback described a tingling sensation at the auricular sites, but no discomfort requiring interruption of therapy.

### 2.3. Treatment results

The patient was systematically evaluated using the hiccups assessment instrument (HAI, 0–10 scale, higher scores indicate more severe symptoms; the HAI score reflects the most severe hiccup intensity experienced within a day) and the Pittsburgh sleep quality index (PSQI) at baseline (before intervention), and on days 1, 2, 3, 4 and 7 after initial intervention.

**Figure 3.**
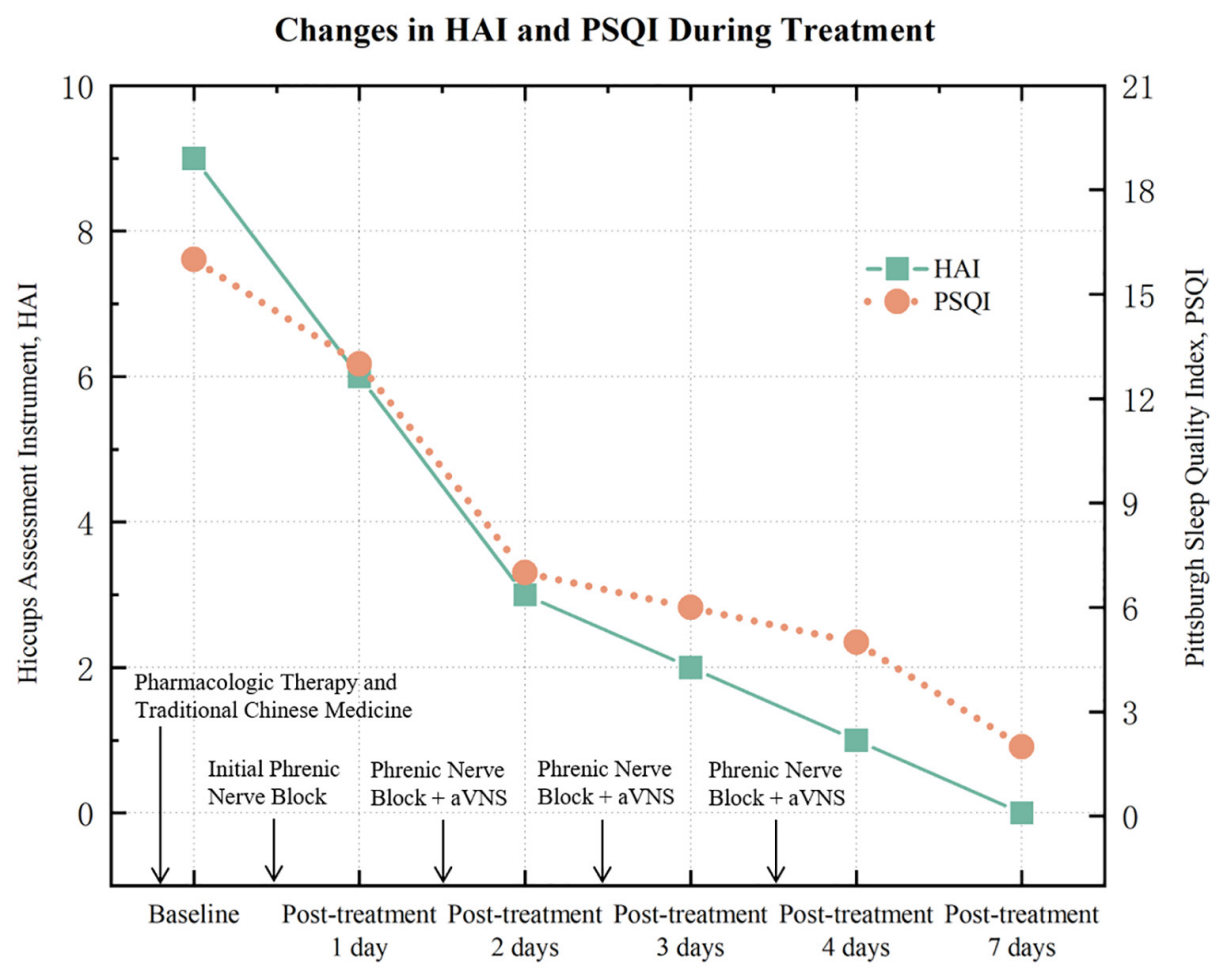
Changes in HAI and PSQI during treatment. aVNS = auricular vagus nerve stimulation, HAI = hiccups assessment instrument, PSQI = Pittsburgh sleep quality index.

At baseline, the patient’s HAI score was 9 and PSQI score was 16, indicating severe hiccups and significant sleep disturbance. After the initial phrenic nerve block (day 1), both scores decreased (HAI = 6, PSQI = 13), with transient hiccup relief lasting about 4 hours, but sleep remained affected. With continued combined therapy (days 2–4), both scores showed a marked downward trend: on day 2, HAI decreased to 3 and PSQI to 7; on day 3, HAI decreased to 2 and PSQI to 6; by day 4, scores further declined to near-normal levels (HAI = 1, PSQI = 5). By day 7, HAI had reached 0 and PSQI reached 2, indicating complete cessation of hiccups and significant improvement in sleep quality (Fig. 3)

No treatment-related complications such as hematoma, vocal cord dysfunction, brachial plexus block, or respiratory compromise were observed. After initiation of combined therapy with ultrasound-guided phrenic nerve block and aVNS, the patient was followed up for 1 month after discharge. At 2-week follow-up, the patient remained symptom-free. By 1 month after discharge, he had resumed normal dietary intake, achieved restorative sleep of 6 to 7 hours per night, and experienced complete resolution of irritability and fatigue. Family members also reported improved social interaction and mood. No delayed complications, treatment-related adverse events, or recurrence of persistent hiccups were identified during follow-up.

## 3. Discussion

Persistent or intractable hiccups are uncommon but can significantly impair quality of life.^[16]^ Epidemiological data suggest that they occur in approximately 0.3 to 0.6% of hospitalized patients, most frequently in elderly males with multiple comorbidities.^[7,16]^ The condition may lead to malnutrition, sleep deprivation, psychological distress, and prolonged hospitalization, thereby aggravating the underlying disease burden. Despite these profound consequences, hiccups are often under-recognized and inadequately managed in clinical practice.^[7]^

Conventional therapies, including pharmacologic agents and nerve blocks, often provide only transient or inadequate relief.^[17]^ This case is unique in demonstrating the combined use of percutaneous aVNS and ultrasound-guided phrenic nerve block, which achieved complete and sustained remission without recurrence, highlighting a promising multimodal strategy for refractory cases.

The pathophysiology of hiccups remains incompletely understood, but evidence indicates an interplay between peripheral afferents, central processing, and neurotransmitter balance.^[5–8]^ Moreover, dysregulation of inhibitory γ-aminobutyric acid transmission or hyperactivity of dopaminergic pathways may lower the threshold for diaphragmatic spasms.^[18]^ Disruption at any level can precipitate symptoms. The most common causes are central nervous system or gastrointestinal disorders, although pulmonary infections may also play a role.^[7,14,19]^ In this patient, pulmonary infection and gastrointestinal disorders were likely the initial triggers. Although these conditions improved with treatment, hiccups persisted, suggesting ongoing peripheral neural hypersensitivity. Neurological examination and central nervous system imaging were normal, making central pathology less likely.

The auricular branch of the vagus nerve is one of the few peripheral cutaneous branches of the vagus, with dense innervation in the cymba and cavum conchae.^[20,21]^ Study has shown that dual-site stimulation in these regions provides optimal efficacy, comfort, and robust neural activation, significantly enhancing activity of brainstem vagal nuclei.^[21]^ By stimulating these areas, aVNS modulates vagal afferent projections to the nucleus tractus solitarius and central autonomic networks, thereby influencing the central components of the hiccup reflex arc.^[20]^ In addition, aVNS may regulate neurotransmitter release within these nuclei (e.g., γ-aminobutyricacid, norepinephrine, acetylcholine), enhancing or inhibiting specific pathways and promoting neuroplasticity.^[22]^ As a novel, noninvasive neuromodulatory technique, aVNS has been applied in the management of conditions such as epilepsy, depression, stroke recovery, and Alzheimer disease.^[23]^ Prior studies indicate that aVNS can regulate neurotransmitter release, reduce central excitability, and improve gastrointestinal function and sleep, making it a rational therapy for persistent or intractable hiccups.^[24,25]^

Phrenic nerve block interrupts efferent signaling to the diaphragm and can provide immediate relief, but its effect is limited by the duration of local anesthetics.^[12,13]^ In our patient, hiccups recurred within hours after the initial block, consistent with previous reports. The addition of aVNS provided central neuromodulation to sustain symptom control, suggesting a synergistic effect between peripheral blockade and central modulation.

Compared with implantable vagus nerve or phrenic nerve stimulation, percutaneous aVNS and ultrasound-guided phrenic nerve block offer safer, less invasive, and more accessible alternatives. Implantable devices, although effective in some cases, require surgical implantation, are costly, and carry risks of lead migration, infection, and poor long-term tolerance.^[12,13,20,23]^ By contrast, both percutaneous aVNS and ultrasound-guided phrenic nerve block can be performed at the bedside and repeated as needed, which enhances feasibility in routine clinical care. In this case, efficacy was objectively assessed using the HAI and PSQI, demonstrating complete resolution of hiccups and significant improvement in sleep quality. Nevertheless, limitations must be acknowledged. This is a single-patient report with a relatively short follow-up period of 1 month after discharge, and large-scale data are lacking. Further prospective studies and randomized controlled trials are warranted to confirm efficacy, optimize stimulation parameters, and define the role of this multimodal approach in managing persistent or intractable hiccups.

## 4. Conclusion

In summary, this case demonstrates that combining ultrasound-guided phrenic nerve block with percutaneous aVNS can provide safe, effective, and sustained relief for persistent hiccups unresponsive to conventional therapies. This multimodal, minimally invasive approach simultaneously modulates afferent vagal input, interrupts efferent phrenic output, and influences central processing within the hiccup reflex arc, thereby offering a rational and feasible therapeutic option. Further prospective studies are warranted to validate its efficacy, optimize stimulation parameters, and explore broader clinical applications.

## Acknowledgements

The authors thank the patient and his family for their cooperation and consent in sharing the details of this case.

## Author contributions

**Conceptualization:** Qinyue Luo, Guipeng Wu.

**Data curation:** Guipeng Wu.

**Formal analysis:** Qinyue Luo.

**Investigation:** Chuangang Shi.

**Supervision:** Ran Wang, Xiaoqing Xu.

**Visualization:** Qinyue Luo, Ran Wang.

**Writing** – **original draft:** Qinyue Luo.

**Writing** – **review & editing:** Qinyue Luo, Guipeng Wu, Chuangang Shi, Zongfeng Guo, Jin Wang, Ran Wang, Xiaoqing Xu.
